# Quantification of the Whole Lymph Node Vasculature Based on Tomography of the Vessel Corrosion Casts

**DOI:** 10.1038/s41598-019-49055-7

**Published:** 2019-09-16

**Authors:** M. Jafarnejad, A. Z. Ismail, D. Duarte, C. Vyas, A. Ghahramani, D. C. Zawieja, C. Lo Celso, G. Poologasundarampillai, J. E. Moore

**Affiliations:** 1 Department of Biomedical Engineering, Johns Hopkins University School of Medicine, Baltimore, Maryland 21205 USA; 2 Department of Bioengineering, Imperial College London, London, SW7 2AZ UK; 3 Department of Life Sciences, Imperial College London, London, SW7 2AZ UK; 4 The School of Mechanical, Aerospace and Civil Engineering, University of Manchester, Manchester, M13 9PL UK; 5 The Francis Crick Institute, 1 Midland Road, London, NW1 1AT UK; 6 Department of Medical Physiology, Texas A&M Health Science Center, Temple, Texas, 76504 USA; 7 The Francis Crick Institute, 1 Midland Road, London, NW1 1AT UK; 8 School of Dentistry, University of Birmingham, Birmingham, B5 7EG UK

**Keywords:** Lymphatic system, Immune system

## Abstract

Lymph nodes (LN) are crucial for immune function, and comprise an important interface between the blood and lymphatic systems. Blood vessels (BV) in LN are highly specialized, featuring high endothelial venules across which most of the resident lymphocytes crossed. Previous measurements of overall lymph and BV flow rates demonstrated that fluid also crosses BV walls, and that this is important for immune function. However, the spatial distribution of the BV in LN has not been quantified to the degree necessary to analyse the distribution of transmural fluid movement. In this study, we seek to quantify the spatial localization of LNBV, and to predict fluid movement across BV walls. MicroCT imaging of murine popliteal LN showed that capillaries were responsible for approximately 75% of the BV wall surface area, and that this was mostly distributed around the periphery of the node. We then modelled blood flow through the BV to obtain spatially resolved hydrostatic pressures, which were then combined with Starling’s law to predict transmural flow. Much of the total 10 nL/min transmural flow (under normal conditions) was concentrated in the periphery, corresponding closely with surface area distribution. These results provide important insights into the inner workings of LN, and provide a basis for further exploration of the role of LN flow patterns in normal and pathological functions.

## Introduction

Lymphatics have been recognised both as a fluid balancing system and an immunological control system. The primary role of the lymphatics is to collect the fluid that is exuded from blood capillaries to the interstitium and return it to the blood stream^1^. In addition to regulating tissue fluid balance, the lymphatics serve as a major transport route for immune cells and interstitial macromolecules. Before the fluid collected from the interstitium is returned to the blood, pathogens must be neutralised by specialised cells in the lymph nodes (LN)^1–3^. These are secondary lymphoid organs of the lymphatic system that are distributed throughout the body, containing high densities of B, T, and other immune cells that neutralise harmful, foreign particles and cancer cells^4–6^.

LN have a highly elaborate structure-functional organisation with various sub-compartments orchestrating specific immune responses. The crosstalk between the hematopoietic and stromal cell compartments plays a pivotal role in developing adaptive immunity^7,8^. LN blood vessels (BV) are highly specialised in facilitating T cell entry to LN^7^. Approximately 90% of T cells in the LN arrived there by migrating across the walls of high endothelial venules (HEV), which have primarily been observed in secondary lymphatic organs^4,6^. There is also an important exchange of fluid across the BV walls^9^. In an elegant series of experiments, Guyton and colleagues demonstrated that the direction and amount of flow can be modulated by local hemodynamic and oncotic pressure differences^9–12^. The movement of fluid across these BV walls can be modelled with the Starling equation^13,14^. There are several reasons why the spatial distribution of this fluid movement could be important for both fluid balance and immune function. The migration of dendritic cells and macrophages into and through the T cell region depends on the distributions of chemokines such as CCL21^15^, which are modulated through a combination of fluid-flow dependent advection, diffusion, binding dynamics, and cell-mediated actions^16^. Metastatic tumour cells express chemokine receptors that regulate their migration into the lymphatic system^2,17,18^, and are transported to the draining LN. Recent studies have demonstrated that metastatic cells migrate toward and into LN BV^19,20^. Also, the direct transport of small antigens is dependent on lymph flow patterns in the T cell region^21^. The local direction of fluid movement will therefore directly or indirectly determine which regions of the LN are exposed to pathogens as well as cancer cells.

The spatial distribution of surface area for exchange of fluid and solutes with BV in LN is a primary factor in determining the amount of local fluid movement^22–24^. Namely, it is important to quantify both the location and diameter of these vessels, which requires a high degree of spatial resolution and contrast. Several attempts have been made at quantifying the LN vasculature. Kelch *et al*. utilized confocal-based imaging platform called extended-volume imaging system to scan and quantify BV in a mouse mesenteric LN^25^. Although this technique provided detailed information on the mesenteric LN vasculature, the time required to process a single node (weeks) hinders the investigation of multiple LNs. Kumar *et al*. employed optical projected tomography and light sheet microscopy to image high-endothelial venules in optically cleared mouse popliteal LNs^26^. The efficiency of this method is acceptable to investigate multiple LNs, but it lacks the resolution to capture the smaller vessels and capillaries that are likely the primary routes of fluid exchange.

**Figure 1:**
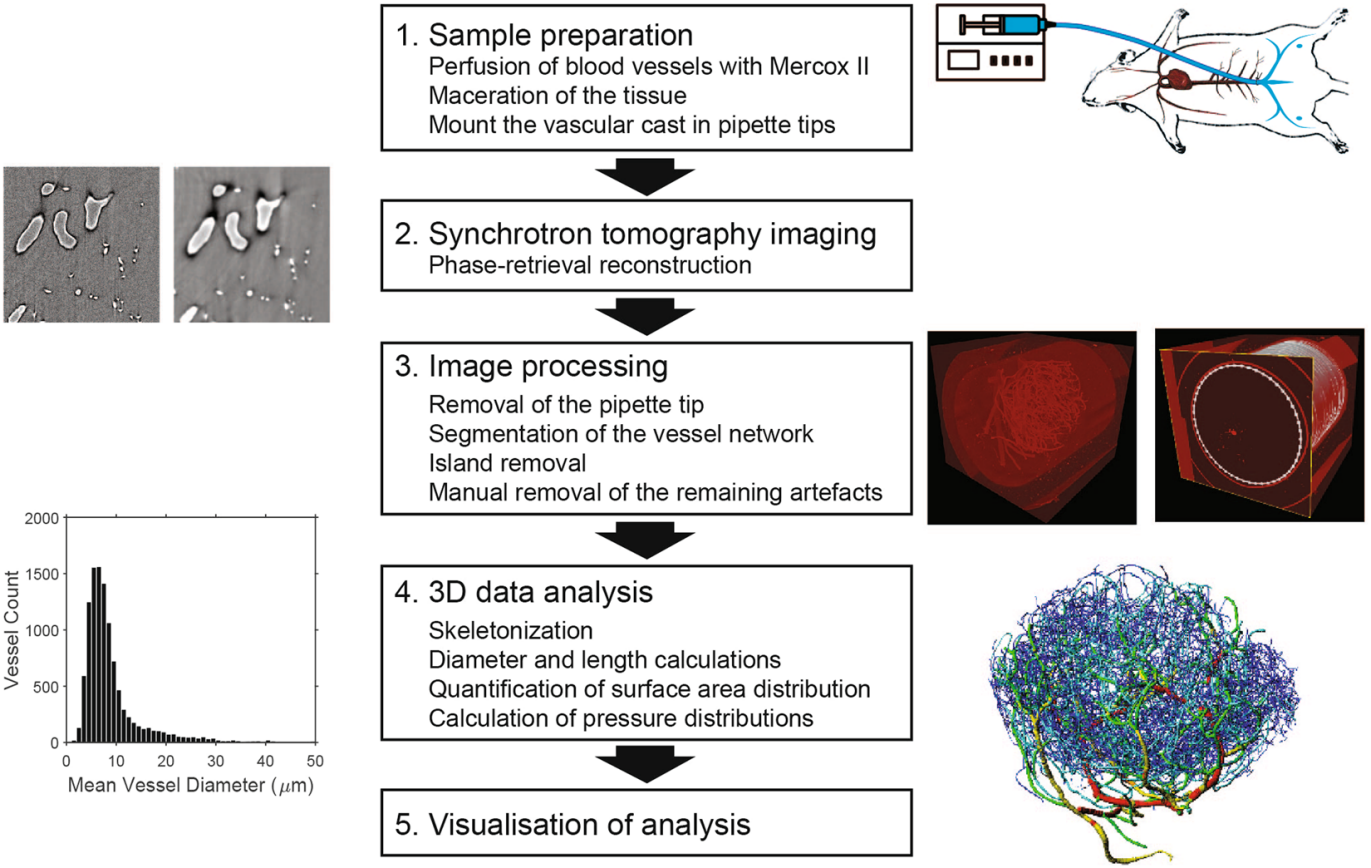
Workflow of vascular casting, tomography and image processing. The blood in LN vasculature was flushed out and replaced with Mercox II resin to cast the lumen of the vasculature. The LN was then surgically excised and placed in a pipette tip before dissolving the tissue with potassium hydroxide (1). The freeze-dried samples were scanned with high-resolution synchrotron tomography and the radiographs were reconstructed into stack images using a phase-retrieval algorithm (2). The images were pre-processed by removing the pipette tip image using a cone crop before intensity-based segmentation and manual artefact processing (3). The binary data were then skeletonized and diameters and length of the vessels as well as the surface area density were calculated. Pressures and velocities of blood flow were estimated in each vessel based on an assumption of Poiseuille flow (4). The results were visualized with Imaris and further parameters quantified and plotted with Matlab (5).

We have previously demonstrated the importance of total LN BV surface area on fluid exchange and intranodal lymph flow^22^, which directly modulate the chemokine gradients in the LN that are essential for T and dendritic cell entry, and effective adaptive immune response^16^. In this study, we aim to establish a corrosion casting technique in combination with phase-contrast synchrotron micro-computed tomography (µCT) to quantify the details of LN vascular structure with the emphasis on quantification of surface area distribution (Fig. 1).

## Results

**Figure 2:**
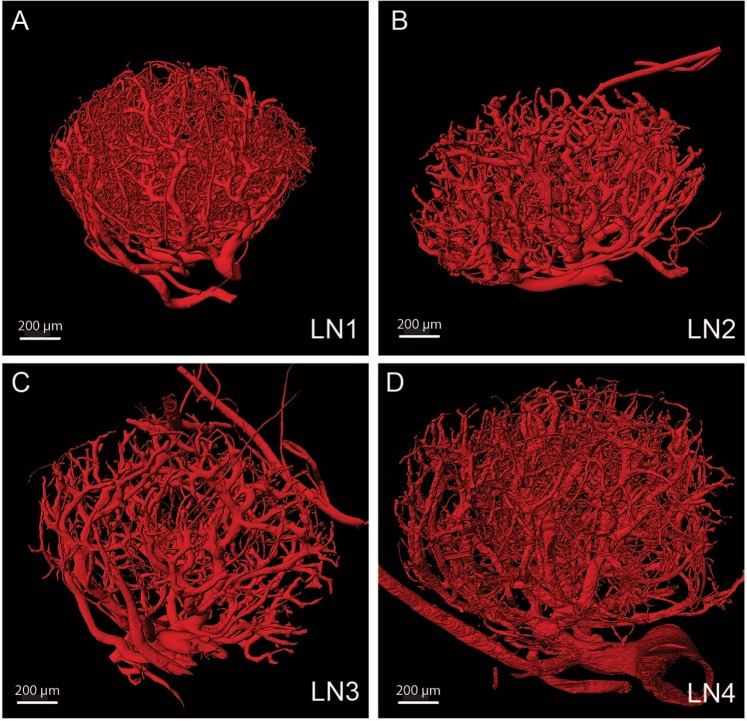
High-resolution 3D images of four mouse LNs. 3D representation of four LN datasets shows the quality of vascular casts. The degree by which the capillaries are preserved varies between the cases. LN1-LN4 (**A**–**D**) are referenced in the rest of the manuscript. Scale bars: 200 µm (**A**–**D**).

**Figure 3:**
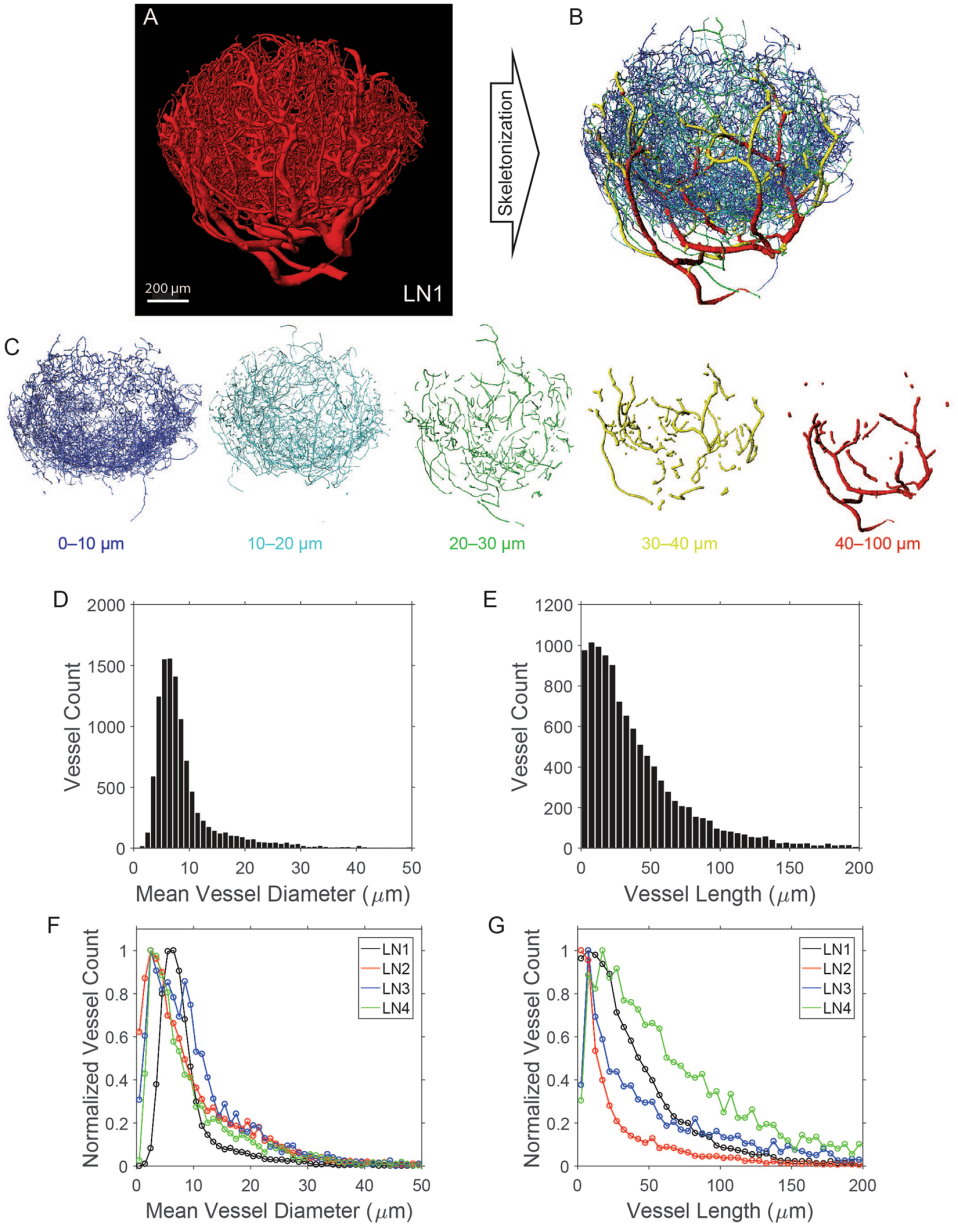
Analysis of vessel length and diameter distribution in the vascular networks. Based on the 3D segmented images of the LN vasculature (**A**) the binary images were skeletonized and the length and diameter of the vessels were calculated. The vasculature was then reconstructed (**B**) with colors corresponding to each diameter category (**C**). Skeletonization was used to quantify vessel characteristics such as diameter (**D**) and length (**E**). Distributions are shown for the node shown in (**B**) in histograms with bins size of 1 µm for diameter (**C**) and 5 µm for length (**D**). Finally, we have performed the analysis for the other three dataset and the distribution of diameters (**G**) and lengths (**H**) show similar expected trends between the datasets. Scale bar: 200 µm (**A**).

The sample preparation and imaging protocols were successful in capturing details down to the capillary level in BV of four mouse LN (Fig. 2) that varied in overall nominal diameter of 1.0–1.4 mm. BV diameters ranged from 82 μm (one of the exiting venules) down to 4 μm (Table 1 and Fig. 3). With the definition of an individual BV being the segment between two branch points, the mean vessel diameter was 9.7 ± 0.7 μm, indicating that most of the branching occurred in the smallest vessels. The total number of BV ranged from 2,447 to 10,765, which showed a mostly inverse but inconsistent relationship with the number of branching points, mean vessel length and vessel density (Table 1). This indicates a substantial variety in BV branching patterns amongst the 4 LN. The BV occupied from 3.2% to 10.1% of the total LN volume, with approximately 60% of that coming from the capillaries in the case of LN1. Node-specific results presented below are all from LN1.

**Table 1:** LN BV network parameters. A “vessel” is defined as the segment between two branching points.

	LN1	LN2	LN3	LN4
Number of Vessels	10,765	6,480	2,447	3,931
Number of Branching Points	6,546	3,451	1,202	3,692
Mean Vessel Diameter (µm)	8.98	9.31	10.27	10.35
Mean Vessel Length (µm)	40.4	36.7	66	76.2
Network Length (cm)	43.5	23.8	16.2	30
Network Volume (mm^3^)	0.06	0.042	0.073	0.051
Network Surface Area (mm^2^)	14.9	9.5	11.3	17.9
Surface Area Density (µm^2^/µm^3^)	0.0156	0.00937	0.0115	0.0112
Fraction of the Total LN Volume (%)	10.1%	4.2%	7.1%	3.2%
Vessel Density (vessels/mm^3^)	11,272	6,420	2,499	2,462
Diameter of Feeding Arteriole (µm)	48.1	28.4	26.6	34.7
Diameter of Main Vein (µm)	63.4	56.4	58.2	82

**Figure 4:**
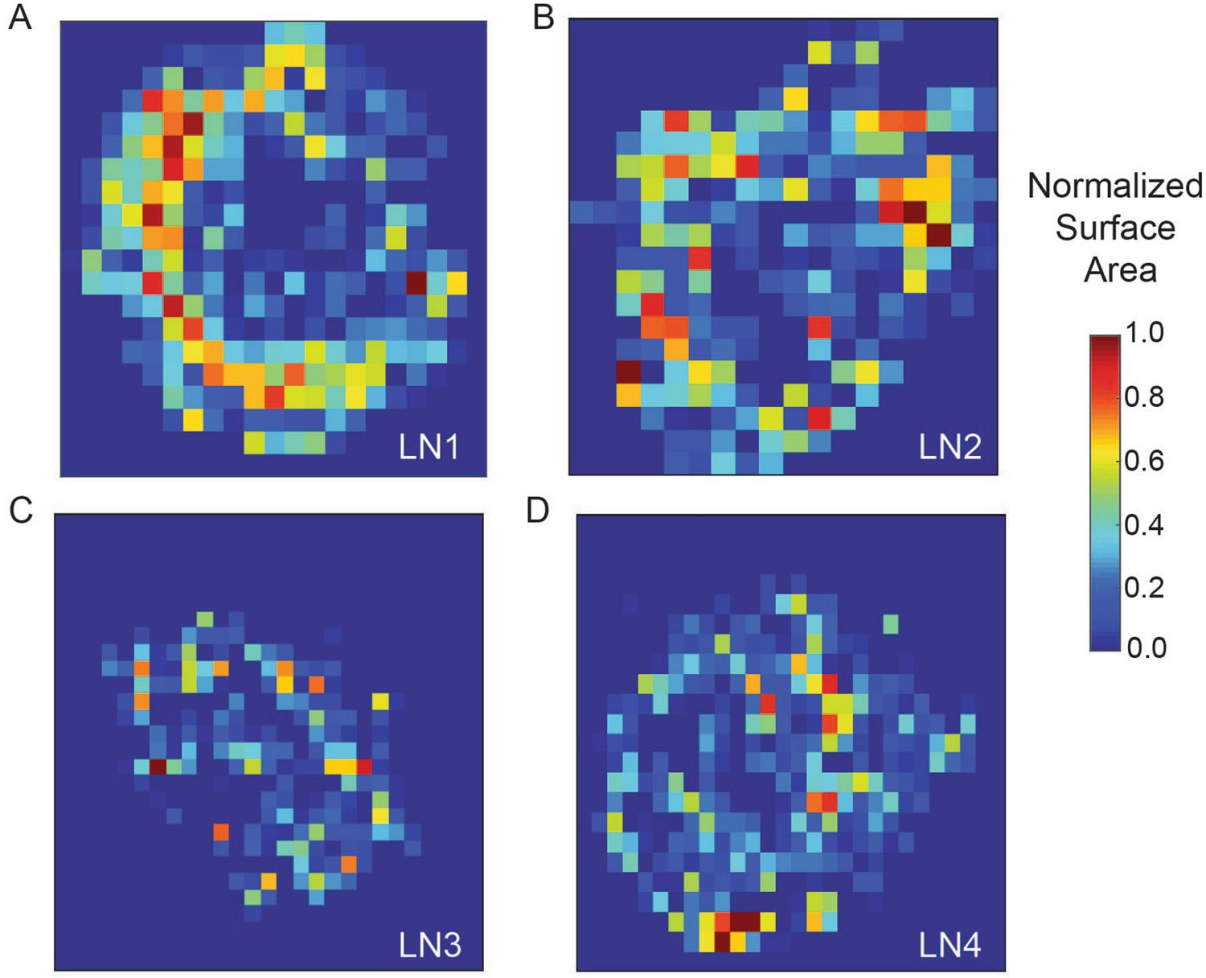
Analysis of the surface area distribution in the vascular networks. The datasets were divided in 80 by 80 by 80 pixel boxes and surface area was calculated within each box and normalized surface area is shown for all four LNs (**A**–**D**). The heatmaps represent the mid plane for all the LNs. Vessels appeared more densely distributed in the periphery of the LN.

The total BV surface area available for fluid exchange with the lymphatic compartment was 13.4 ± 3.7 mm^2^, with an overall density of 0.012 ± 0.003 μm^−1^. The surface area was distributed in a highly inhomogeneous manner, with most of it concentrated near the LN periphery (Figs 4 and S2). The area variation is illustrated by summing the surface area over voxels of varying size. The peripheral concentration was evident at all voxel sizes. The distribution is intimately tied to the concentration of capillaries around the periphery, with 76% of the surface area coming from that class of vessels (Table 2).

**Table 2:** Contribution of different vessel types to surface area, and flow in LN1.

	Arteries	Veins	Capillaries
Volume (% of total)	13.9	25.9	60.2
Surface Area (% of total)	10.0	13.8	76.2
Flow rate (% of total)*	3.0	16.7	80.3

*P_lymph_ = 20 mmHg for calculation of the transmural flow rate.

**Figure 5:**
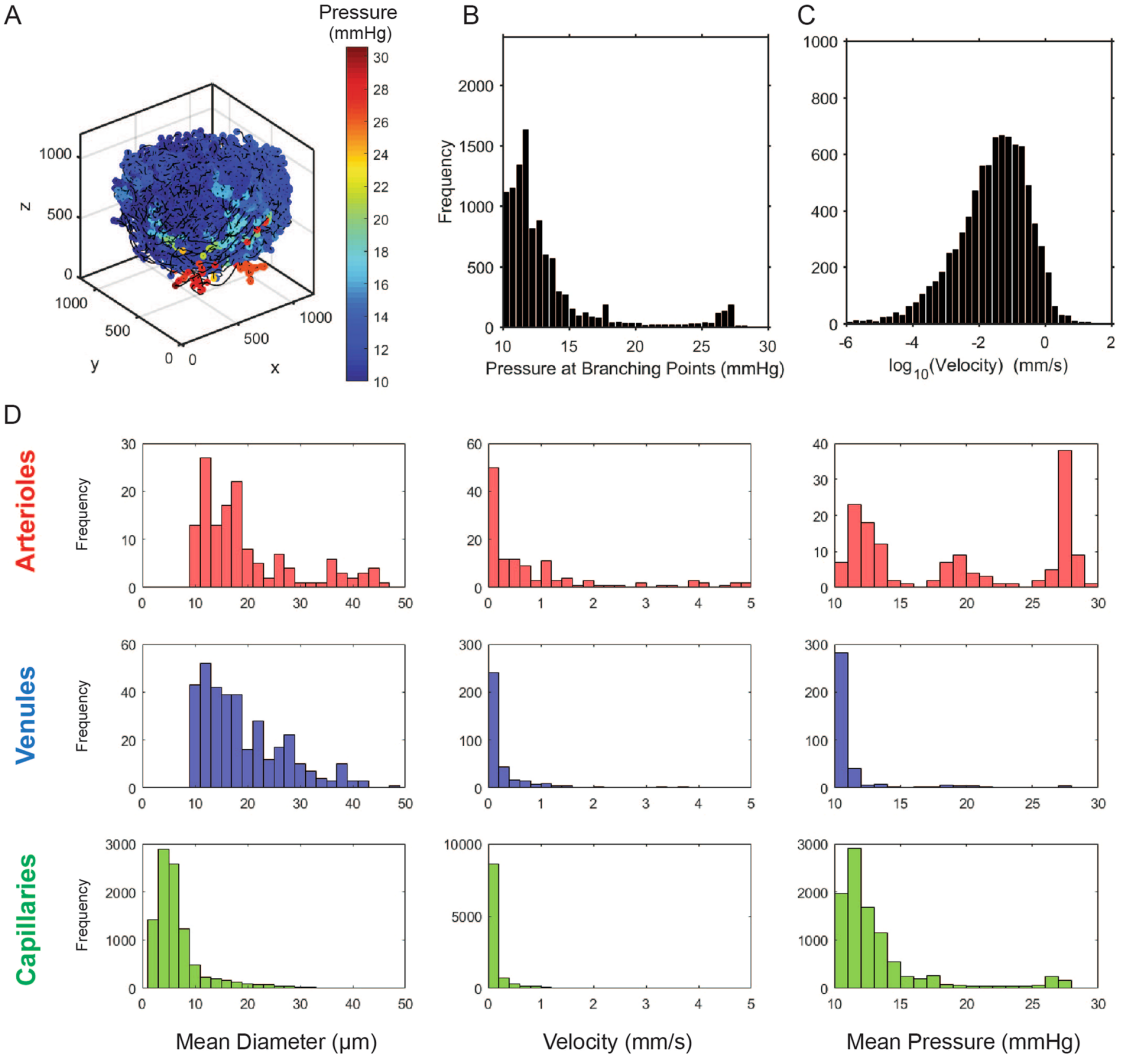
Distribution of blood pressure and velocities in the vascular network. The blood pressure in the LN vessels was skewed towards the vein pressure represented by LN being mostly blue in panel A. The distribution showed distinct sets of vessels with a peak at high pressures (~25 mmHg) that was associated with arterioles, vessels with pressures near LN vein pressure (assumed to be 10 mmHg) that was associated with venules and especially high-endothelial venules, and capillaries in between these two sets of vessels. Distribution of velocities in the LN vessels was close to a log normal distribution (**C**), with higher velocity in larger vessels such as arterioles and large venules. The distribution of mean vessel diameter, velocity and pressure are shown for different subsets of vessels separated into three categories of arterioles (red), venules (blue) an capillaries (green).

Full 3D characterization of the BV network was also used to estimate hemodynamic pressures along the network, which in part determine the flux across the BV wall. Using a diameter-dependent apparent viscosity model^27^, the total pressure drop across the network was 19.5 mmHg, which along with our assumed venule exit pressure of 10 mmHg and a total flow rate of 0.22 µL/min, gave an entry arteriolar pressure of 29.5 mmHg. The pressure distribution across the network provides further illustration of its complexity, indicating that the network is not an ordered, straight-forward progression of branches into progressively smaller branches. Pressures in vessels characterized as arteries varied across the whole range, and there were a few vessels characterized as capillaries that showed arteriole-level pressures (Fig. 5). This was due to the fact that there were vessels more than 10 generations of branches past the entering arteriole that were classified as capillaries, but >30 μm in diameter. Because of the predominant number of capillaries, the hemodynamic velocity distributions were concentrated at the lower values (<1 mm/s). Higher values of velocity were found in the arterioles, which are generally smaller in diameter than their corresponding venules.

**Figure 6:**
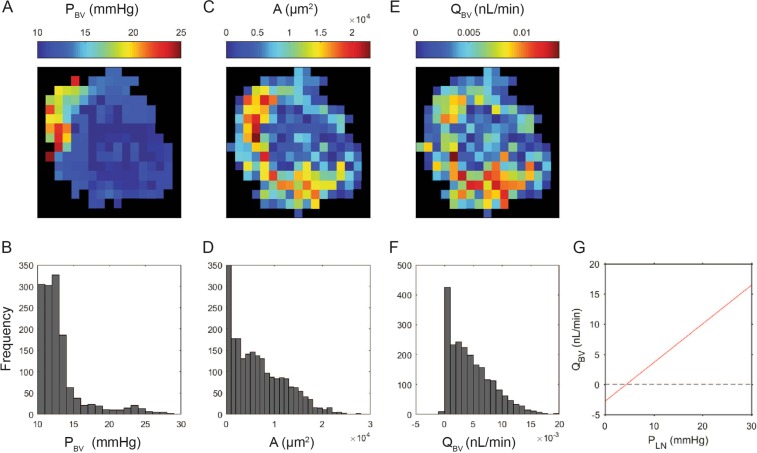
Distribution of parameters important in blood lymph fluid exchange based on the Starling equation. Distribution of the blood pressure ((**A**) mid section, and (**B**) whole LN) shows that the blood pressure in most of regions of the LN is close to the LN venous pressure (assumed to be 10 mmHg). Distribution of surface area ((**C**) mid section, and (**D**) whole LN) appears exponential. Distribution of fluid flow from LN to BV that linearly correlates with pressure multiplied by surface area ((**E**) mid section, and (**F**) whole LN) shows a non-uniform distribution with higher values in the periphery of the LN. The contours in the top panels (A, C and E) are for a single slice in the middle of the LN, and the histograms in the bottom panels (B, D, and F) are for the whole LN. The LN was divided into 100 by 100 by 100 pixel boxes (81 × 81 × 81 µm^3^) for these analyses.

**Figure 7:**
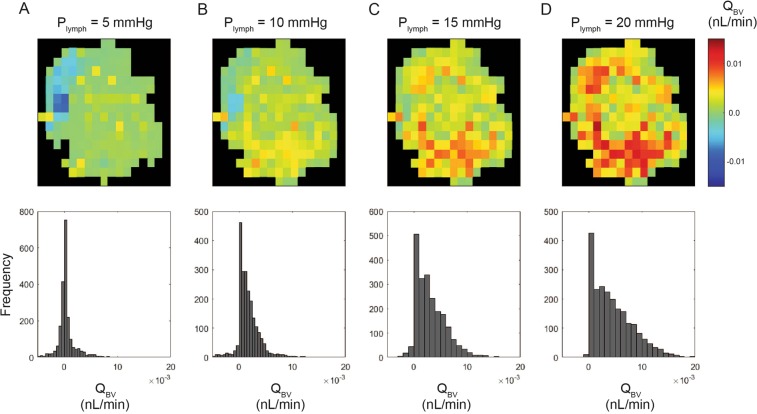
Effect of LN pressure on blood lymph fluid exchange flow.Mean pressure in lymphatic channels of LN linearly affects the amount of fluid exchange from LN to BV and changes the distribution and pattern of exchange flow. At low P_lymph_ (**A**) there are fluid exchange in both directions based on the location in the LN, and increasing P_lymph_ shifts the fluid transport in favor of LN to BV (B-D). The contours in the top panels are for a single slice in the middle of the LN, and the histograms in the bottom panels are the results for the whole LN. The LN was divided into 100 by 100 by 100 pixel boxes (81 × 81 × 81 µm^3^) for these analyses.

The distribution of flow across the BV wall (taken as positive from the lymph compartment to the blood) was primarily determined by the distribution of surface area. This corresponded to the concentration of the surface area in the capillary beds around the LN periphery, as described above. The distribution of the transmural flow mapped well with the surface area, but less so with the BV pressure (Fig. 6). With a uniform lymphatic compartment pressure P_lymph_ of 20 mmHg, flow across the walls of individual vessels ranged from slightly negative 10^−3^ nL/min to positive 20 × 10^−3^ nL/min. The sum total of transmural flow throughout the LN was 10 nL/min. Increasing P_lymph_ to 30 mmHg increased transmural flow to 15 nL/min. Net flow into the BV was zero at a P_lymph_ of 4 mmHg, and below that value the net flow was from blood to lymph. The histograms of flow distribution in Fig. 7 illustrate that changing P_lymph_ does not result in a simple shifting of flow values, due to the uneven distribution of surface area amongst the BV.

## Discussion

In this study we present a novel technique to image and quantify the distribution of the surface area of BV in a whole and healthy mouse popliteal LN. Each LN includes an important interface between the blood and lymphatic systems. LN BV are specialized structures, featuring HEVs, across which immune cells are supplied to the LN^7,26^. These BV also present a potentially crucial location for metastatic cells to migrate into the blood stream, where they can then be transported to secondary tumour sites^19,20^. The transmural movement of fluid has been demonstrated to vary sensitively with the driving pressure gradients within the physiologic range^10,11^. Our study aimed to provide spatially-resolved 3D data on the distribution of these BV, with the eventual goal of understanding LN transport phenomena down to the cell-level scale. We found that BV are non-uniformly distributed in LN, with most of the surface area available for fluid transport being located around the LN periphery.

The overall volume percentage of BV compares well with observations of other well-vascularized murine tissues^28–31^. In hind-limb skeletal muscle, BV accounted for 12 ± 5% of the tissue volume, increasing to 32 ± 13% with VEGF treatment^28^. Nebuloni *et al*.^29^ found similar results in the hind-limb of 2–6% BV volume, and further specified that 1% of the total volume was occupied by BV >500 µm in diameter, and <0.5% was occupied by vessels with diameters between 100 and 500 µm. Mohan Das *et al*.^30^ quantified BV volume as 7.6% of kidney tissue and 10.4% of liver tissue using microCT images with a 20 µm voxel size.

Our results showed that capillaries were responsible for approximately 75% of the surface area of the BV network, which totaled 13.4 ± 3.7 mm^2^. This was nearly three times the surface area of the 1 mm diameter node itself. In the example shown in Fig. 5, veins accounted for 14% of the BV surface area, while arteries accounted for 10%. The spatial distribution of the BV surface area, assessed by summing the surface areas within voxels, showed high concentrations around the periphery of the node.

Because the capillaries represented the majority of the surface area available for fluid transport, most of the flow across BV walls was predicted to occur in these peripheral regions. Changing the overall lymphatic pressure had strong effects on both the distribution and direction of the fluid flow across the BV wall. When the lymph pressure is high, fluid moves mainly from the lymph compartment through the capillaries into the blood. When lymph pressure is reduced below approximately 4 mmHg, the overall flow direction changes to shift fluid from blood to lymph, and most of this flow occurs across the walls of the larger arteries. The range of total transmural flows (up to 15 nL/min) corresponds well with our previous computational flow model, which assumed BV were uniformly distributed in the T cell cortex^22^. The shifts in transmural flow show the same trends as those demonstrated in canine LN by Adair and Guyton^10,11^.

Inflammatory or immune responses cause an increase in LN size, along with lymphangiogenesis and angiogenesis^32–37^. This swelling affects the vascular density, as shown by light-sheet microscopy of fluorescently labeled HEVs of the mouse LN^26,38^. Although recent advancements in tissue optical clearance and staining have shown promising results^39,40^, the study of the whole LN microvascular networks has been limited and burdensome^25^. The LN vasculature imaging technique utilized here is a scalable approach that allows timely study of the LN BV remodeling under multiple inflammatory and treatment conditions. Previously, µCT imaging has been used to image vascular networks in muscle tissue^28^, liver^41^, kidney^42^, breast cancer tumors^31,43^, and in the whole animal^30^. The level of detail in the final images is highly dependent on the sample preparation, surrounding tissue artefacts and the resolution limits of the scanner. Our images were gathered at the state-of-the-art Diamond Synchrotron Facility at Harwell. The high energy of the synchrotron beam allowed rapid high-resolution scans that took about 3–5 minutes for a whole LN, compared to scan times of approximately hours to a day for the high-resolution stand-alone µCT scanners capable of sub-micron voxel sizes.

While the incorporation of fully 3D BV geometry provides significant new insights into LN fluid transport, it is important to recognize the major limitations of this study. The vessel diameters were calculated based on the voxel-based volume quantification and the length of the centerline from skeletonized vessels that are prone to inherent errors of the techniques, although these errors should be relatively small because of the voxel size in this study (pixel size of 0.81 µm). The radioopacity of Mercox II was similar to water and the surrounding tissue meaning that without any contrast, it was impossible to resolve the LN vasculature. Addition of radio-opaque agents (e.g. phosphotungstic acid (PTA), iodine, and radio-opaque nano-particles) to Mercox II, interfered with the polymerization and significantly increased the viscosity of the casting polymer solution, which resulted in unsatisfactory casting of the microvessels. Additionally, staining of the surrounding tissue by PTA led to numerous difficulties with uneven staining and development of bubbles in the sample during the scan due to the high-energy of the X-ray beam (Supplementary Fig. 1A). Furthermore, the prepared vessel cast samples were very brittle, and required delicate handling. We are continuing to refine our imaging protocols to alleviate these limitations. For the analysis, both the hydrostatic and oncotic pressures in the lymph compartment were assumed to be constant, whereas *in vivo* these will both vary spatially as lymph moves through the LN and is concentrated due to fluid movement into BV. Our previous modeling study predicted a hydrostatic pressure loss of approximately 1 mmHg across the entire lymph node^22^, so this effect should be minor compared to the incorporation of BV pressure variation as was done here. The hydrostatic pressure distribution in the BV was calculated based on the non-Newtonian apparent viscosity model of Pries^27^, which should provide greater fidelity than a Newtonian model. However, a comparison of these two models (not shown) showed minimal effects on transmural flow. Due to the lack of available experimental data, we have assumed that all BV walls exhibit the same hydraulic conductivity. It is likely that capillaries are more porous, which would further shift the fluid movement noted here towards those vessels. Given their unique structure and importance in immune cell trafficking, it would be highly beneficial to obtain an estimate of HEV wall hydraulic conductivity.

In summary, we developed a corrosion casting technique in combination with high-resolution synchrotron µCT to visualize the BV network of mouse popliteal LN. This study quantified the BV surface area density as well as its spatial distribution in the LN, which allows us to more accurately estimate the fluid exchange between the blood and lymph compartments in the LN. Further computational models with accurate representation of surface area distribution are necessary to determine the role of local fluid exchange to blood on chemokine gradients and immune cell migration in the LN. Changes in LNBV structure and resulting LN flow patterns due to immune reaction and cancer are also topics worthy of further investigation.

## Methods

### Animals and sample preparation

All experiments were undertaken with the approval of the Imperial College’s Animal Ethics Committee and were in accordance with its guidelines and the requirements of the United Kingdom Home Office regulations (ASPA 1986). The workflow of vascular casting, µCT scanning and image analysis is illustrated in Fig. 1. All animals were intraveneously (I.V.) injected with 60 µL of 800 units/ml of heparin in Phosphate Buffered Saline (PBS) through the tail vein to prevent blood coagulation 10 min prior to euthanasia with a lethal dose of sodium pentobarbital I.V. Then, the abdominal aorta was exposed after an anterior incision in the median plane. The abdominal aorta was then cannulated in the caudal direction, at a point distal to the renal artery branches. The cannula was fixed by suturing around the vessel. The vessels downstream to this point were perfused with the following solutions prior to resin perfusion: 1) PBS with 100 units/ml of heparin to flush out coagulations, 2) 4% paraformaldehyde (PFA) in PBS to fix the vessels and avoid subsequent leakage. During the first perfusion with PBS and heparin, a small incision was made in the vena cava to facilitate outflow. A resin preparation containing 0.1 g of benzoyl peroxide (Sigma) in 5 ml of blue Mercox II resin (Ladd research industries) was then perfused into the vessels. This resin was administered carefully through the abdominal aorta with a syringe pump. Initially a high flow rate (~1 ml/min) was used to quickly fill the vessels; the flow rate was subsequently reduced by one-half every minute to counter the increasing pressure (a result of the rising viscosity caused by the resin polymerization inside the vessels). Once the resin had partially hardened (about 10 min after the start of the resin perfusion), LN were carefully and surgically extracted and placed in PBS overnight at 4 °C to allow the hardening of the resin to complete. The LN were then macerated by placing the tissue in 7.5% potassium hydroxide (KOH) for 24 hr at 60 °C to dissolve the tissue and reveal the cast. The quality of the casts was evaluated by stereo or bright-field microscope. The casts that appeared to have a good coverage of the LN vasculature were freeze-dried inside a 200 µl pipette tip and used for synchrotron µCT scans. This technique was chosen and refined after considering a few approaches in preparing LN vasculature samples for µCT (Fig. S1).

### Synchrotron micro-computed tomography

X-ray micro tomography experiments were carried out at the Diamond Manchester Branchline (i13-2)^44^ at Diamond Light Source, Didcot, UK. Polychromatic X-ray beams with energy in the range of 8–35 kV filtered with C and Al were used to probe the samples. X-rays transmitted through the samples produce visible light on striking a scintillator (500 µm thick CdWO_4_) positioned in line with the beam and the sample. The detector was placed 25 mm from the sample to generate in-line phase contrast while minimising edge blurring. The produced visible light was magnified using 8x objectives and captured using a sCMOS camera (PCO edge 5.5) with a detector array of 2560 × 2160 pixels giving an effective pixel size of 0.81 µm. In total, 3601X-ray projections were captured at 20 fps while the sample was continuously rotated from 0 to 180°. The 3D datasets were reconstructed using a filtered-back projection algorithm^45^ before and after the application of Paganin filter^46,47^.

### Quantification of blood vascular network

The quantification of the vessel network was done using Imaris image processing software (Bitplane, Belfast, United Kingdom) (Fig. 2). To prepare the image stack for importation to Imaris, the image stack file was converted to 8-bit, noise was filtered out using a (3 × 3 × 3) median filter, and the pipette image and other artefacts were subtracted out. A thresholding segmentation using Otsu’s method was used to segment BV from the surrounding tissue^48^. Using the built-in ‘skeletonisation’ algorithm in Imaris, the centrelines of the vessel network, and the points (branching and end) were identified. A vessel is defined as the segment that connects two points (branching or end). The algorithm then calculated the vessel length based on the pathlength of the centreline between the two connected points. The average vessel diameter was calculated based on the volume of the vessel (product of the number of pixels in the vessel and the volume of a pixel that is 0.53 µm3) divided by the vessel length. Next, the surface area distribution of the BV network was calculated using a custom algorithm implemented in Matlab 2017b. The image stack was sectioned into cubic blocks of different sizes, and the surface area of the vessel segments within each block was summed (using the ‘patch’ and ‘isosurface’ functions in Matlab (Figs S3, S4). The total surface area was then calculated from the sum of the surface areas in the blocks (Table 1).

### Pressure and velocity distribution of vascular network

Based on an electrical circuit analogy and assuming laminar, fully-developed flow, the pressure at all junctions and resulting flow were calculated using an empirically-derived non-Newtonian apparent viscosity for blood^27^. For boundary conditions, we assumed a constant flow of 0.22 µL/min through the feeding artery to result in a wall shear stress of 10 dyn/cm2, and constant pressure of 10 mmHg at the main vein leaving the LN. The flow through all the other end points (truncations due to lack of identification of the continuation of the vessel) was assumed to be zero.

### Classification of LNBVs

Vessels up to ten generations of branching from the main arteriole and venule that were more than 10 µm in diameter were classified as arteries and veins, respectively. The rest of the LNBVs were assumed to be capillaries.

## Supplementary information

Supplementary Info

## Data Availability

The Lymph Node image datasets generated during the current study, are available in the figshare repository 10.6084/m9.figshare.8289869 ^49^.
