# Supplementary material for: Quantification of the Whole Lymph Node Vasculature Based on Tomography of the Vessel Corrosion Casts
Source: Sci Rep. 2019 Sep 16;9:13380. doi: 10.1038/s41598-019-49055-7 (PMC6746739; doi:10.1038/s41598-019-49055-7)
Supplement: Supplementary file 1 — Supplementary Info [file 41598_2019_49055_MOESM1_ESM.docx]

# Quantification of the Whole Lymph Node Vasculature Based on Tomography of the Vessel Corrosion Casts

# M. Jafarnejad, A. Z. Ismail, D. Duarte, C. Vyas, A. Ghahramani, D.C. Zawieja, C. Lo Celso, G. Poologasundarampillai, J.E. Moore Jr.

**Supplementary Figures**


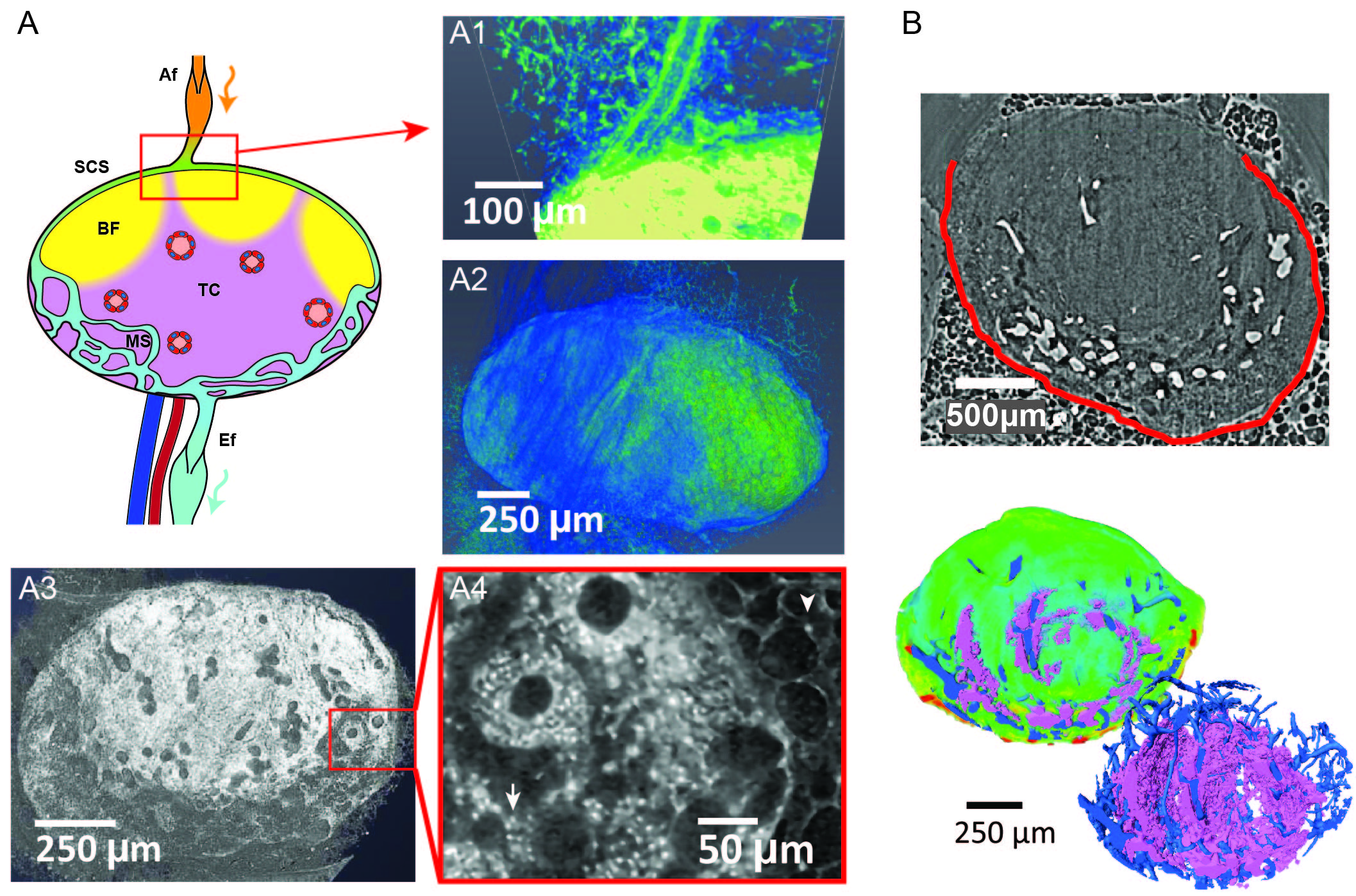


Figure S1. Alternative approaches in visualization of LN structure using synchrotron X-ray 3D tomography (sCT).

Schematic of the mice PLN is used to guide the images from the sCT approaches that can be employed to study structure and function of the LN (A1: afferent vessel, A2 whole lymph node, A3: single slice from entire mice LN showing both lymph space and blood vessels, A4: zoomed in image of the red box in A3 showing single and clusters of cells both inside (white arrow) and outside (arrow head) the LN). In the first approach (A), the LNs were casted with resin and subsequently the tissue was stained with PTA (tissue-binding contrast agent), while the secondary approach is to perfuse the vessels with resin that contains a contrast agent. Approach in A was more informative but the segmentation is extremely difficult. Although approach in B can provide more information than the primary approach in this article (casting with complete removal of the tissue), It provides less information than approach A and it is very difficult to add contrast agent to the resin without increasing the viscosity and hence reducing the resin penetration (top panel: a single slice before any pre-processing with BV in high contrast, tissue in medium contrast and lymph space in low contrast. Bottom panel: sCT images of a PLN digitally cut in half showing the tissue (green), BV (blue) and lymph space (pink) segmented and rendered in 3D.)


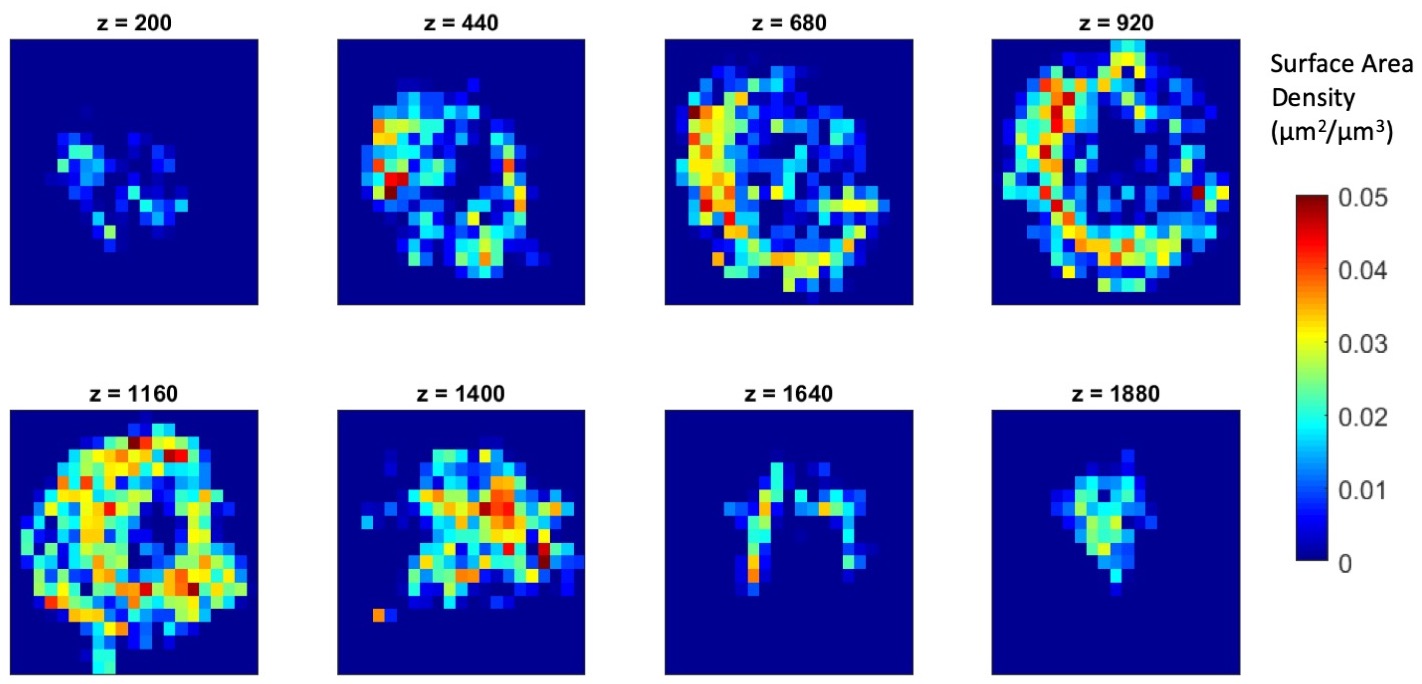


Figure S2. Surface area density at different z locations. Each slice is 80 pixels thick and the middle of the slice is located at the z pixel stated on top of each panel.


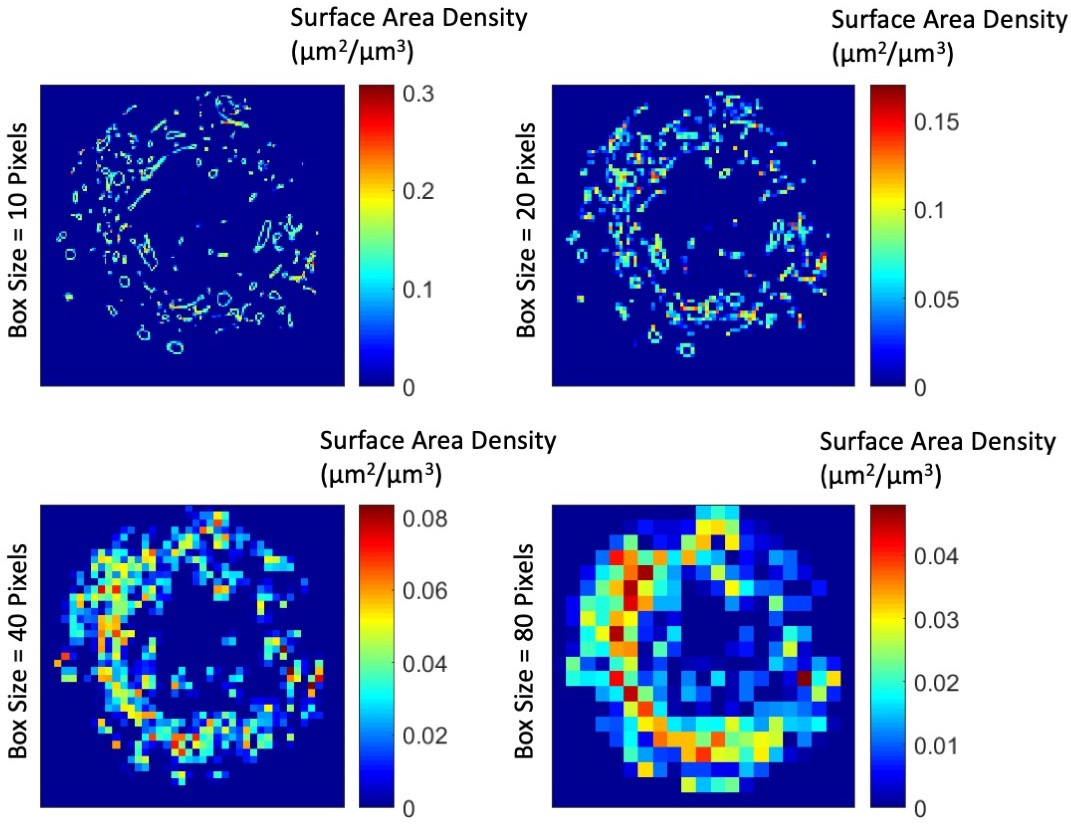


Figure S3. Surface area of the vasculature when varying the size of the box.


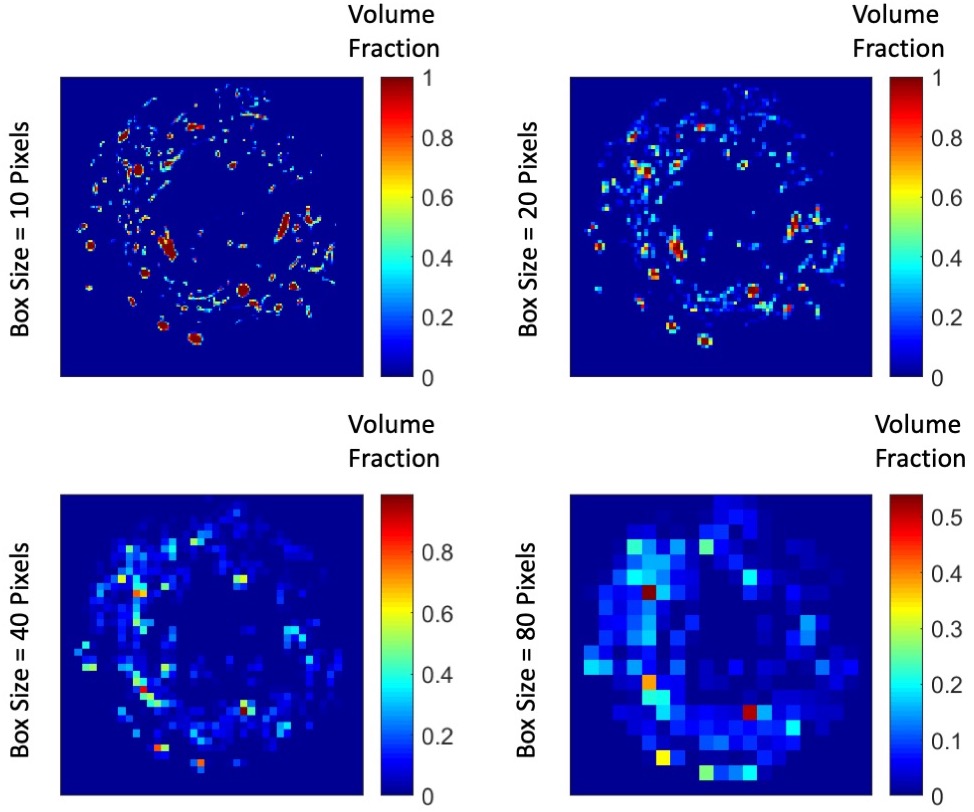


Figure S4. Fraction of the volume of the box occupied by vasculature.
